# Anatomical, Pathological, and Therapeutic Researches upon the Disease Known under the Name of Gastro-Enterite, &c.

**Published:** 1838-04

**Authors:** 


Art. VII.—*Anatomical, Pathological, and Therapeutic Researches upon the Disease known under the Name of Gastro-Enterite; Putrid, Adynamic, Ataxic, or Typhoid Fever, &c.; compared with the most common Acute Disease.* By P. C. A. Louis, m.d. &c. *Translated from the French, by* H. J. Bowditch, m.d.—*Boston,* 1836. Two Vols. 8vo. pp. 395, 462.

These are two remarkably handsome volumes printed at Boston, and, if they meet with a satisfactory sale in America, it will shew that there is much zeal for pathological information amongst our transatlantic brethren. The original work of M. Louis is an application of his numerical method on a large scale, and alone is sufficient to hand his name to posterity as a most careful, indefatigable, and judicious observer of facts; but it is the reverse of popular: it requires diligent study, and will remain a standard book of reference. It differs from the generality of French medical works in being dry and unattractive; for, in general, our neighbours manage to impart some of their liveliness to their serious labours: it also differs from too many in one object which M. Louis tells us he had in view, "not to write one single useless phrase." It is full of minutely reported cases and rigorous deductions. M. Louis says that, between 1822 and 1827, he collected 138 cases of typhus fever, fifty of which were fatal. He analyzed all; and, in order to ascertain which of the lesions found in the patients who died were peculiar to typhus, he compared them with the alterations found as consequences of other acute diseases in eighty-three subjects, whose histories he learned. He did the same with the symptoms of those affected with typhus fever, or with any other acute affection, which terminated either favorably or fatally; so that he has analyzed the diseased changes of the viscera of 133 subjects, and the symptoms of nearly 900. When it is recollected that this labour was undertaken for the investigation of fever only, that M. Louis has taken equal pains with phthisis, and still continues his exertions as a clinical teacher, it will excite no surprise to those who are not acquainted with M. Louis to learn that, in accurate diagnosis, he has no rival. The motto M. Louis prefixes to his work, from Rousseau's "Emile," is given, we presume, as an authority for the numerical method; but it contains, we think, the fallacy on which the numerical method, when carried out to treatment, exists. "Je sais que la vérité est dans les choses, et non dans mon esprit qui les juge, et que moins je mets du mien dans les jugemens que j'en porte, plus je suis sûr d'approcher de la vérité." (*Emile.*) It may be literally true that the truth resides in the facts themselves, and not in the mind of the persons judging them; but, when Rousseau adds, that the less he introduces what is merely his own into the judgments he pronounces, the more he approaches the truth, he altogether overlooks the fact that it is by his mind that he judges, and that the rectitude of the decision greatly depends on his own mental acuteness; for the correctness of any deduction from probable evidence, which requires what we call judgment, depends materially on the correct powers of judging which the individual possesses who investigates. For instance, Harvey stated that he discovered the circulation of the blood by reflecting on the use of the valves: now, the existence of the valves had been known long before his time, but it wanted the reflective powers of a Harvey to discover the great truth which these valves suggested. Probably Rousseau himself merely intended to say that, in judging correctly, we should free ourselves from prejudices.

The translation, wherever we have made the comparison, is faithful, but it is too literal: it reads like a translation, which no translation ought to do. Dr. Bowditch's dedication to the memory of his friend, James Jackson, jun., and his enthusiasm for his teacher, give us a very favorable impression of himself; and we trust that he may be able, at some future time, to complete the design he announces of giving some account of typhus fever as it appears in Boston, which he thinks is identical with the typhoid fever of Paris: with great consistency, however, he does not fix his opinion in this point until he can prove it numerically.

